# Diseases of Lachrymal Apparatus

**Published:** 1874-03-15

**Authors:** L. L. Lambert

**Affiliations:** Galesburg, Ill.


### MEDICAL EXAMINER.

A

*Semi-Monthly Journal of Medical Sciences.*

EDITED BY N. S. DAVIS, M.D., AND F. H. DAVIS, M.D.

NO. VI. CHICAGO, MARCH 15, 1874. VOL. XV.

### Original Communications.

√

### DISEASES OF LACHRYMAL APPARATUS.

A PAPER READ BEFORE THE MILITARY TRACT MEDICAL SOCIETY, JAN. 13, 1874, BY L. L. LAMBERT, M.D., GALESBURG, ILL.

MR. PRESIDENT AND GENTLEMEN: I have thought I might with advantage invite your attention to some of the diseases affecting the lachrymal apparatus. I shall not enlarge upon their pathology or diagnosis, but make some general remarks, and some more special, in reference to their treatment.

Diseases of the different parts, one or more, going to make up this apparatus are very common, and receive but too slight attention from the profession generally. I do not think this obtains from a direct want of knowledge of these diseases, and of their appropriate treatment, but, seemingly, only from the indirect manner in which patients are put off by the physician when they call for treatment.

Excuse the diversion; but I would remark here that there is a greater percentage of persons inconvenienced by some derangement of the organs of hearing, than there is of those suffering from disease of any other one organism; and you, gentlemen, are aware of the unconcerned attention paid to ear-patients generally.

In this last class of affections, and in those of the lachrymal apparatus, were all patients to receive, when first they call upon their physician for advice, that faithful attention which nearly all know so well how to favor them with, then would the patient be relieved, would be spared years of inconvenience, and permanent injury averted, which, coming on, remains as an advertisement of the negligence of the physician, who, with the ability, did not see anything very bright or brilliant to stimulate him to make curative use of his practical knowledge in the direction of these uninviting diseases.

Physicians do have a distaste, as a general thing, for the treatment of nearly all of the diseases of the ear and eye. This is not right. And it is this distaste which so often excuses them—with themselves—for turning patients away with a very casual examination, and with just as casual a prescription. I must say that I think it is such attention, given and received, which causes to be developed a great majority of the worst cases met with in these classes of disease.

But I would confine myself to lachrymal diseases, not alone enumerating names, but would allude to the symptoms and course of some, and speak of the importance of their early treatment; and, if you please, exhibit for your inspection a selection of instruments and appliances I use for their treatment in my practice.

In reference to dacryocystitis, or inflammation of the sac, and to blennorrhœa of sac or mucocele, I need only say that the first-mentioned is an *active* inflammation, while the latter, mucocele, is a passive inflammation of the sac. Either disease may precede, or be followed by, the other, while in the presence of either are developed much the same effects, and much the same course of symptoms. With the symptoms and diagnosis of these diseases you are all familiar. In either case you will notice the small tumor just below the inner canthus, filled, in the case of dacryocystitis, with pus, and, in the case of mucocele, with a gray, viscid mucous of a light color. In the first case you may fear the formation of a fistulous opening, and a discharge of the pus through the anterior wall of the sac, and through the cutis. In the second case, mucocele, this danger is not so much to be apprehended as that of the escape of the contents of the sac through the puncta, which occurs the more readily since these contents are fluid.

In these cases, when they first occur, or after, the indications for treatment are, first, to provide a ready means of escape for the pus from the sac. This is best accomplished by slitting the lower canaliculus, and possibly also the upper one, well into the sac. For this purpose there have been various knives constructed by Bowman, Weber, and others; but the best instrument is the scissors, such as I show you in this selection. They are much the easier instrument to manipulate, produce the least pain, and are more certain to divide the canaliculus well into the sac. The canaliculus, divided, provides a ready passage for the tears into the sac, and thus the annoying epiphora is relieved, which continuing, the tears flowing over the lower lid, irritate the skin, and this contracts and produces a partial ectropion, which further distorts the lids and puncta; and thus is added a quite serious complication to an already bad disease.

Secondly, ascertain immediately whether there is a stricture, partial or complete, of the nasal duct. This is readily ascertained by the use of Anel's eye-syringe, which I show you, and which is fitted with gold points for insertion into the puncta, and can be used for cleansing the sac and duct, even before the canaliculus is divided. If the fluid readily passes down into the nostril, there will be no need of using the probe. If it does not, then use the probe. For this purpose I have made choice of a small whalebone, or, better still, a small lead probe; either of which will not allow of sufficient force to produce false passages, and, again, are so pliable as to readily adapt themselves to any curvature they may pass, and in the case of the lead one, when carefully removed, it at once indicates to you the curves to be sought and used, in case a style is found necessary.

In all cases in which water can be made to pass through the duct, I deprecate the use of the probe or style; for, too often, strictures are thus formed where none existed before. But in case we meet with a firm obstruction in the duct, it must be broken down. The lead probe may be found to fail, and you must resort to the silver probe. But this will seldom occur; and in these cases I recommend the immediate use of the style. Now, in my practice I use those made by myself, of lead, and of such size as I may determine in each case, usually commencing with a small size. But whatever style you use at first, never leave it in the passage longer than twenty-four or thirty-six hours, as the existing inflammation is most prone, in all cases, to assume an erysipelatous character, and will soon develop into active and real erysipelas. At first, then, frequently remove the style, and perfectly cleanse the passage. The wearing of an appropriate style is much the better treatment than frequent probings. After the subsidence of all symptoms of active inflammation, the style can be left in for a longer time. But before this, I would caution you against leaving it in longer than twenty-four or thirty-six hours, and to frequently cleanse the passage. I use as astringents for cleansing the sac and duct, a two or four grain solution of zinci sulph., cupri sulph., acetate of lead, or alum; or, far better than either, a solution of carbolic acid, from one-half to two grains, to one ounce of water.

As a general remark, I would add, if there is nasal catarrh, treat this, that the inflammation may not again extend up the duct and again reproduce the stricture, or the inflammation of the sac, or the mucocele.

I think that, in nine cases out of ten, if the patient were treated with Anel's eye-syringe, and the frequent free use of the carbolic solution, when he first noticed any difficulty with the tear-passages, stricture would not result: and if at all, would readily yield to this simple treatment; and in quite obstinate cases, a persistence in this treatment will often reward you with the most pleasant and permanent results, probably without having recourse to any other operative treatment than a slitting up of the canaliculus, or, may be, the canaliculi.

In case it should be necessary to wear a style, this, as well as the probe, should always be inserted through the divided canaliculus, following the natural passage.

The practice of puncturing the sac and passing the probe or style through this puncture, has fallen into well-merited disuse.

There is a common practice now of dividing the stricture in several directions, by the use of a knife devised by Dr. Stilling, two of which I show you in this selection of instruments,

The knife is passed into the sac through the divided canaliculus, and thence into the duct, and the stricture divided in at least three or four directions. This practice is a good one, as it does away with the use of the probe and style, and the patient is freed from the inconvenience and annoyance of wearing a style for a long time. But, to compensate for this relief, he has to undergo quite a formidable surgical operation.

In the hands of energetic, skilkful, and experienced physicians, this practice finds much favor, as it accomplishes good cures in a short time.

One more fact I would notice, and that is this, that, in the case of strictures treated with the probe alone, by division, and more especially when the style is used, the after-treatment is of the utmost importance to secure a good result. In fact, it amounts to but little—the mere breaking down of a stricture; and where most fail is in not giving the patient that careful after-treatment of which I have spoken.

